# The Regulation of the Complementary Health Sector: General Public’s Knowledge of Complementary Medicine-Related Quality Assurance and Consumer Protection

**DOI:** 10.34172/ijhpm.2021.56

**Published:** 2021-06-09

**Authors:** David Sibbritt, Wenbo Peng, Jon Wardle, Cameron Stewart, Paul Komesaroff, Jon Adams

**Affiliations:** ^1^Faculty of Health, University of Technology Sydney, Sydney, NSW, Australia.; ^2^Sydney Law School, University of Sydney, Sydney, NSW, Australia.; ^3^Faculty of Medicine, Nursing and Health Sciences, Monash University, Melbourne, VIC, Australia.

**Keywords:** Regulation, Complementary Medicine, Quality Assurance, Consumer Protection, Australia

## Abstract

**Background:** Complementary medicine (CM) use is a ubiquitous aspect of an increasingly consumer-driven model of healthcare delivery and plays an increasingly prominent role in the Australian health sector. Yet there is limited empirical research investigating the quality and integrity of protections for consumers in Australia. The aim of this study is to help address this gap in knowledge by exploring how members of the public engage with protection mechanisms related to CM use.

**Methods:** This study utilised a cross-sectional online survey to recruit a sample of 1132 Australian adults aged 18 and over. Purposive convenience sampling was used to recruit participants from an existing database of Australian adults who had expressed interest in participating in research.

**Results:** The majority of the participants (64.0%) had visited a CM practitioner in their lifetime. However, a minority of participants (36.9%) indicated they would feel confident in knowing where to complain if something went wrong with the treatment they received from a CM practitioner. Most participants (74.7%) had used a CM product in their lifetime. Specifically, 32.3% had ‘ever’ used an herbal product and 69.9% had ‘ever’ used a nutritional supplement. However, a minority of participants (32.7%) indicated they would feel confident knowing where to complain if something went wrong with a herbal or nutritional supplement they used. Most participants indicated a lack of knowledge about how CM practitioners and CM products are regulated in Australia.

**Conclusion:** The findings of this study clearly highlight a concerning lack of knowledge by CM patients and consumers regarding the regulation of CM in Australia. From a policy perspective, it is necessary to seek proactive approaches that target complaint-related knowledge of the CM patients and consumers through education and advocacy efforts.

## Background

**Key Messages**

**Implications for policy makers**

- Use of complementary medicine (CM) is a ubiquitous aspect of an increasingly consumer-driven model of healthcare delivery and plays an increasingly prominent role in the health-seeking of Australians, with 75% of study participants having used a CM product in their lifetime and 64% having visited a CM practitioner in their lifetime.
- Most of the study participants indicated a lack of knowledge about how CM practitioners and CM products are regulated in Australia and relatedly, most of the study participants did not feel confident in knowing where to complain if something went wrong with the treatment they had received from a CM practitioner and/or if something went wrong with a herbal or nutritional supplement they had used.
- Amid this complex regulatory and legal landscape, very little is known about the regulatory risks associated with what CM practitioners and manufacturers actually do in their practice, and the impact on consumers; thus impeding the development of effective and workable regulatory and legislative provisions.
- It is necessary for policy-makers to seek proactive approaches that target complaint-related knowledge of the CM patients and consumers through education and advocacy efforts.

**Implications for the public**

The findings of this study clearly highlight a concerning lack of knowledge by complementary medicine (CM) patients and consumers regarding the regulation of CM in Australia. With only three of the CM professions in Australia being fully incorporated into the national health practitioner registration system and the remainder of the CM professions being self-regulated or currently beyond regulation, it is not surprising that patients find it difficult to identify avenues for directing complaints against CM practitioners. This study also clearly highlights the need for education and advocacy efforts to improve complaint-related knowledge of the CM patients and consumers.

The use of complementary medicine (CM) – practices not traditionally associated with biomedical practice or the medical curriculum^1^ – is a ubiquitous aspect of an increasingly consumer-driven model of healthcare delivery and plays an increasingly prominent role in the health-seeking of Australians. Recent research shows 63.1% of the general Australian population has used CM with almost half of them have used CM supplements^2^ and approximately $AUD 9.3 billion was spent for CM supplements and other unsubsidised/over-the-counter drugs in Australia.^3^ To date, research around CM issues has focused primarily on the reasons for the choice of CM over ‘conventional’ medicine, the nature and extent of CM use, and/or the scientific evidence in relation to its efficacy and effectiveness for a variety of clinical conditions.^1^ Yet there is limited empirical research investigating the quality and integrity of protections for consumers in light of the high levels of use of CM and its significant role in and impact on healthcare in Australia.^4^

 CM plays a substantial but often hidden role in primary and secondary healthcare in Australia^5,6^ and poses similar direct (eg, malpractice) and indirect (eg, failure to appropriately refer through misdiagnosis or monopolisation of care) risks to patients in common with other forms of health service.^7^ However, CM also presents specific challenges for patients, healthcare professionals and those managing the healthcare system. For example, CM often involves promotion and distribution through online sources, use which goes undetected, and products or practices that may not always conform to standards expected of regulated health services and products.^8^ There is both broad public support for CM regulation and evidence that regulation can effectively manage the risks associated with CM use.^9^ Nevertheless, the highly contentious and politicised nature of CM often results in a hesitance to regulate these practices by governments.^10^

 Current Australian health regulation does not address the CM market in a comprehensive way.^10,11^ Only chiropractors, osteopaths and Chinese medicine practitioners are fully incorporated into the national health practitioner registration system (ie, the Australian Health Practitioner Regulation Agency) alongside medical and allied health practitioners, but other CM practitioners such as naturopaths, herbalists, and massage therapists are at best self-regulated (voluntary membership of professional associations). However, even if CM practitioners are not even self-regulated, they must adhere to the National Code of Conduct* for Health Care Workers*. Although, only the States of New South Wales, Victoria, Queensland and South Australia (Tasmania not yet commenced) have created laws to enact the Code. The purpose of the National Code of Conduct for Health Care Workers is to protect the public by setting minimum standards of conduct and practice for all unregistered healthcare workers, including CM practitioner, who provide a health service. It sets national standards against which disciplinary action can be taken and if necessary a prohibition order issued. Similarly, some CM products are ‘registered’ with the Therapeutic Goods Administration (TGA) while most are only ‘listed’ (requiring a much lower evidence base). Some CM – such as medicinal drinks or powders – are marketed as foods rather than therapeutic goods and may not be regulated as a therapeutic good at all.

 There are some similarities between Australia and other countries, in terms of the regulation of CM practitioners and products. In its global report on traditional and complementary medicine (T&CM), the World Health Organization (WHO) described the national or state level laws or regulations for T&CM for 179 (of the 194) WHO Member States.^12^ As at 2018, 109 Member States reported the presence of a legal or regulatory framework for T&CM. In many Member States, the national laws and regulations for T&CM are integrated into the national drug or medicine laws, while for other Member States the T&CM legal framework is the responsibility of state, provincial or territorial jurisdictions, and regulation varies from jurisdiction to jurisdiction. For most Member States, the regulation of herbal medicines came under the scope of the food and drug regulatory authorities.

 Despite the increasingly significant role of CM use in Australian healthcare, it remains unclear how regulatory and consumer protections interface with CM care or public use of CM. In direct response to this gap, the aim of the study reported here is to explore how members of the public engage with protection mechanisms related to CM use.

## Methods

###  Study Design and Participants

 This study utilised a cross-sectional online survey to recruit a sample of 1132 Australian adults aged 18 and over, during the period August-September 2019. Purposive convenience sampling was used to recruit participants from an existing database of Australian adults who had expressed interest in participating in research. The database is administered by a commercial survey company* Qualtrics *(https://www.qualtrics.com). The survey took an average of 15 minutes to finish, and participants received a small financial compensation on completion. Free and informed consent of all participants has been obtained.

###  Measurements

 This online survey covered five domains, including items with regards to participants’ demographic characteristics, the use of CM, the understanding of the regulation of CM practitioners and products, the confidence in knowing where to complain CM use, and the perceptions of the TGA numbering of CM supplements.

 Demographic questions included gender, age, postcode of residence, the highest level of educational qualification obtained, and employment status. The use of CM was determined via two questions. Participants were asked if they had ever consulted with a range of CM practitioners, including chiropractor, osteopath, Chinese medicine practitioner, massage therapist, naturopath, Western herbalist, homeopath, aromatherapist, yoga therapist, ayurvedic practitioner, and reiki healer. Participants were also asked if they had ever used any CM products, including aromatherapy oils, Western herbal medicine, Chinese herbal medicine, ayurvedic herbal medicine, flower essence, homeopathic remedies, fish oil/krill oil/omega 3, multivitamins, or other vitamins/minerals. The CM practices and products used in the survey were chosen as Australian surveys indicate these are the most popularly used in Australia.^13^

 To obtain insight into the participants’ understanding of the regulation of CM practitioners and products in Australia, participants were asked to indicate the level of regulation they thought applied to each listed CM practitioner (ie, unregulated, self-regulation, government monitored self-regulation, statutory regulation) and each listed CM product (ie, unregulated, registered as foods, a listed medicine, a registered medicine). Participants were also asked to rate, on a 5-point Likert scale, how confident they were in knowing where to complain if something went wrong with the treatment received from a CM practitioner or if something went wrong with a herbal or nutritional supplement. In addition, participants were asked if they had complained about a CM practitioner or a herbal or nutritional supplement they had used to a doctor, CM practitioner or regulatory authority.

 In Australia, medicines approved for supply must display an Australian Register of Therapeutic Goods (ARTG) number. The ARTG number starts with ‘AUST’ and is followed by an ‘R’ or ‘L’ (ie, AUST-R or AUST-L). The ARTG number not only identifies the product but is a guide as to how much examination the product has received before going on sale, including whether the TGA has assessed the medicine for efficacy. Medicines with AUST-R are assessed for efficacy, while those with AUST-L are not.^14^ To obtain insight into the participants’ understanding of the AUST-R and AUST-L numbering in relation to herbal and nutritional supplements, participants were asked whether or not the following statements were true or false: AUST-R nutritional supplements or herbal products are assessed for safety, quality and effectiveness *[true]*; AUST-L nutritional supplements or herbal products are assessed for safety, quality and effectiveness *[false]*; AUST-L nutritional supplements or herbal products can only contain pre-approved low-risk ingredients *[true]*; and AUST-R nutritional supplements or herbal products can only contain pre-approved low-risk ingredients *[false]*.

###  Statistical Analyses

 The software program STATA 14 was used for statistical analyses. Data were analysed using descriptive statistics including frequency and percentage for the categorical variables. Bivariate analyses were conducted using chi-square tests. Logistic regression modelling was undertaken to examine the association between demographic characteristics and participants’ confidence in knowing where to make a complaint if something went wrong with the treatment they received from a CM practitioner and/or a herbal/nutritional supplement they used. Statistical significance was set at *P* = .05.

## Results

 A total of 1132 participants were recruited into the study. Table 1 provides descriptive statistics for each demographic characteristic measured. It can be seen that most participants (58.5%) were male. In addition, most participants (48.2%) were aged under 35 years, with 9.9% aged 65 years and over, and 31.1% having obtained a school only education with 37.7% a university education.

**Table 1:** The Demographic Characteristics of Study Participants

**Characteristic**	**Frequency**	**Percent**
Age group (y)		
18-24	243	21.4
25-34	303	26.8
35-44	203	17.9
45-54	130	11.5
55-64	141	12.5
65 and over	112	9.9
Gender		
Male	470	41.5
Female	662	58.5
State		
Australian Capital Territory	15	1.3
New South Wales	325	28.7
Victoria	285	25.2
Queensland	254	22.4
South Australia	106	9.4
Western Australia	111	9.8
Tasmania	28	2.5
Northern territory	8	0.7
Education		
No formal or high school only	351	31.0
Trade/apprenticeship or certificate/diploma	354	31.3
University	427	37.7
Employment status		
Full-time employed	412	36.4
Part-time employed	322	28.4
Unemployed	398	35.2

###  Complementary Medicine Practitioners

 The majority of the participants (64.0%) had visited a CM practitioner in their lifetime. However, a minority of participants (36.9%) indicated that would feel confident in knowing where to complain if something went wrong with the treatment they received from a CM practitioner. This lack of confidence is accompanied by the participants’ lack of knowledge about how CM practitioners are regulated in Australia. Note, participants were considered to feel confident if they indicated that they either “agree” or “strongly agree” with the statement “if something went wrong with the treatment I received from a complementary medicine practitioner, I would feel confident to know where to complain.”

Table 2 presents the findings from logistic regression models examining the association between demographic characteristics and participants’ confidence in knowing where to make a complaint if something went wrong with the treatment they received from a CM practitioner. The only statistically significant demographic characteristics were gender and age. Specifically, compared to males, females (odds ratio [OR] = 0.46; 95% CI: 0.35, 0.59) were less likely to feel confident to know where to complain. In comparison to the 18-24 years age group, participants from all other age groups were less likely to feel confident to know where to complain (ORs ranging from 0.32 to 0.64).

**Table 2:** Logistic Regression Models Examining the Association Between Demographic Characteristics and Participants’ Confidence in Knowing Where to Make a Complaint if Something Went Wrong With the Treatment They Received From a CM Practitioner and/or a Herbal/Nutritional Supplement They Used

**Demographic Characteristics**	**Treatment From a CM Practitioner**			**Herbal/Nutritional Supplement Used**		
	**Odds Ratio**	**95% CI**	***P*** ** Value**	**Odds Ratio**	**95% CI**	***P*** ** Value**
Gender						
Male	1.0	-		1.0	-	
Female	0.46	0.35, 0.59	<.01	0.52	0.40, 0.68	<.01
Age group						
18-24	1.0	-		1.0	-	
25-34	0.64	0.44, 0.92	.02	0.77	0.53, 1.13	.19
35-44	0.54	0.36, 0.81	<.01	0.69	0.46, 1.05	.08
45-54	0.32	0.20, 0.53	<.01	0.55	0.34, 0.88	.01
55-64	0.37	0.23, 0.59	<.01	0.38	0.23, 0.62	<.01
65 and over	0.46	0.27, 0.76	<.01	0.34	0.19, 0.60	<.01
State						
Australian Capital Territory	1.0	-		1.0	-	
New South Wales	2.48	0.67, 9.11	.17	1.34	0.41, 4.43	.62
Victoria	2.29	0.62, 8.47	.21	1.21	0.37, 4.00	.75
Queensland	1.89	0.51, 7.02	.34	1.19	0.36, 3.95	.89
South Australia	1.86	0.48, 7.16	.37	1.15	0.33, 3.99	.82
Western Australia	2.40	0.63, 9.19	.20	1.29	0.37, 4.43	.69
Tasmania	2.32	0.51, 10.47	.27	1.46	0.35, 6.07	.60
Northern territory	2.08	0.29, 14.84	.46	0.80	0.11, 6.02	.83
Education						
No formal or high school only	1.0	-		1.0	-	
Trade/apprenticeship or certificate/diploma	1.02	0.74, 1.44	.87	1.04	0.74, 1.47	.81
University	1.16	0.83, 1.62	.37	1.19	0.85, 1.67	.31
Employment						
Full-time employed	1.0	-		1.0	-	
Part-time employed	1.03	0.74, 1.43	.81	0.96	0.69, 1.34	.83
Unemployed	0.85	0.61, 1.20	.36	0.77	0.55, 1.09	.14

Abbreviation: CM, complementary medicine.

Table 3 shows that the participants’ ability to correctly identify the form of regulation for each CM practitioner group ranged from 6.8% to 44.9%. Apart from in the case of naturopathy (*P* = .01) and reiki healer (*P* = .01), whether or not a participant consulted the specific CM practitioner did not appear to influence the participants’ ability to correctly identify the form of regulation for each CM practitioner group. Further, of the 724 participants who had visited a CM practitioner in their lifetime, 131 (18.1%) indicated that they had complained to a medical doctorabout how a CM practitioner treated them; 143 (19.8%)had complained to another CM practitioner about how a CM practitioner treated them; and 109 (15.1%) had complained to a regulatory authority about how a CM practitioner treated them.

**Table 3:** Participants Understands of the Regulation of Different CM Practitioner Groups

**Form of Regulation**	**Visited CM Practitioner(s)**				***P*** ** Value**
	**Yes**		**No**		
	**Correctly Identified Form of Regulation**		**Correctly Identified Form of Regulation**		
	**Yes**	**No**	**Yes**	**No**	
	**n (row %)**	**n (row %)**	**n (row %)**	**n (row %)**	
Statutory regulation					
Chiropractic	104 (24.9)	313 (75.1)	144 (20.1)	571 (79.9)	.06
Osteopathic	38 (23.5)	124 (76.5)	210 (21.6)	760 (78.4)	.61
Chinese Medicine	19 (5.4)	330 (94.6)	53 (6.8)	730 (93.2)	.40
Self-regulation					
Massage therapy	220 (42.8)	294 (57.2)	234 (37.9)	384 (62.1)	.09
Naturopathy	110 (44.9)	135 (55.1)	317 (35.7)	570 (64.3)	.01
Western herbal medicine	51 (41.5)	72 (58.5)	375 (37.2)	634 (62.8)	.35
Homeopathic	52 (38.0)	85 (62.0)	367 (36.9)	628 (63.1)	.81
Aromatherapy	61 (42.1)	84 (57.9)	343 (34.8)	644 (65.2)	.09
Yoga therapy	76 (36.5)	132 (63.5)	354 (38.3)	570 (61.7)	.63
Ayurvedic	44 (39.6)	67 (60.4)	336 (32.9)	685 (67.1)	.15
Unregulated					
Reiki healer	65 (43.6)	84 (56.4)	326 (33.2)	657 (66.8)	.01

Abbreviation: CM, complementary medicine.

###  Herbal Products and Nutritional Supplements

 Most participants (74.7%) had used a CM product in their lifetime. Specifically, 32.3% had ‘ever’ used a herbal product and 69.9% had ‘ever’ used a nutritional supplement. However, a minority of participants (32.7%) indicated they would feel confident in knowing where to complain if something went wrong with a herbal or nutritional supplement they used. Table 2 shows that gender and age were significantly associated with confidence in knowing where to complain. Specifically, compared to males, females (OR = 0.52; 95% CI: 0.40, 0.68) were less likely to feel confident to know where to complain. In comparison to the 18-24 years age group, participants aged 45 years or older were less likely to feel confident to know where to complain (ORs ranging from 0.34 to 0.55).

 This lack of confidence in knowing where to complain is reflected in the participants’ lack of knowledge about how herbal and nutritional supplements are regulated in Australia. Table 4 shows that the participants’ ability to correctly identify the form of regulation for each herbal and nutritional supplement ranged from 13.5% to 38.4%. Apart from Chinese herbal medicine (*P* = . 02), Ayurvedic herbal medicine (*P* < .01) and flower essences (*P* < .01), whether or not a participant used the specific herbal or nutritional supplement did not appear to influence the participants’ ability to correctly identify the form of regulation for each herbal or nutritional supplement. Further, of the 845 participants who had used herbal or nutritional supplements in their lifetime, 101 (12.0%) indicated that they had complained to a medical doctorabout a herbal or nutritional supplement they used; 103 (12.2%)had complained to a CM practitioner about a herbal or nutritional supplement they used; and 83 (9.8%) to a regulatory authority about a herbal or nutritional supplement they used.

**Table 4:** Participants Understands of the Regulation of Different Herbal or Nutritional Supplements

**Form of Regulation**	**Used Herbal/Nutritional Supplement(s)**				***P*** ** Value**
	**Yes**		**No**		
	**Correctly Identified Form of Regulation**		**Correctly Identified Form of Regulation**		
	**Yes**	**No**	**Yes**	**No**	
	**n (row %)**	**n (row %)**	**n (row %)**	**n (row %)**	
**Supplement**					
Aromatherapy oils	76 (18.9)	327 (81.1)	148 (20.3)	581 (79.7)	.56
Western herbal medicine	70 (31.1)	155 (68.9)	249 (27.5)	658 (72.5)	.28
Chinese herbal medicine	77 (32.0)	164 (68.0)	219 (24.6)	672 (75.4)	.02
Ayurvedic herbal medicine	43 (38.4)	69 (61.6)	235 (23.0)	785 (77.0)	<.01
Flower essence	45 (24.9)	136 (75.1)	128 (13.5)	823 (86.5)	<.01
Homeopathic remedies	49 (25.0)	147 (75.0)	222 (23.7)	714 (76.3)	.70
Fish oil/krill oil/omega 3	153 (28.4)	386 (71.6)	148 (25.0)	445 (75.0)	.19
Multivitamins	208 (30.2)	481 (69.8)	126 (28.4)	317 (71.6)	.53
Other vitamins/minerals	149 (27.6)	391 (72.4)	173 (29.2)	419 (70.8)	.54

 The participants demonstrated a poor understanding of the implications of an AUST-R or AUST-L number of a herbal product label, with 24.7% correctly identifying AUST-R herbal products as assessed for safety, quality and effectiveness, and 12.6% correctly identifying AUST-L herbal products as not assessed for safety, quality and effectiveness. Further, 19.4% correctly identified AUST-L herbal products as only able to contain pre-approved low-risk ingredients, while 10.3% correctly identified AUST-R herbal products as not containing pre-approved low-risk ingredients. In addition, the participants also demonstrated a poor understanding of the implications of an AUST-R or AUST-L number of a nutritional supplements label, with 25.6% correctly identifying AUST-R nutritional supplements as assessed for safety, quality and effectiveness, and 10.9% correctly identifying AUST-L nutritional supplements as not assessed for safety, quality and effectiveness. Further, 18.5% correctly identified AUST-L nutritional supplements as only able to contain pre-approved low-risk ingredients, while 9.7% correctly identified AUST-R nutritional supplements as not containing pre-approved low-risk ingredients.

## Discussion

 This study provides the first examination of how members of the public engage with and the extent to which they understand protection mechanisms related to CM use in Australia. Our analyses revealed two key findings. First, most participants did not feel confident in knowing where to complain if something went wrong with the treatment they had received from a CM practitioner and/or if something went wrong with a herbal or nutritional supplement they had used. Second, this lack of confidence appears to be reflected in the participants’ lack of knowledge about how CM practitioners and/or how herbal and nutritional supplements are regulated in Australia.

###  Confidence in Knowing Where to Direct Complaints Against CM Practitioners

 With only three of the CM professions in Australia being fully incorporated into the national health practitioner registration system and the remainder of the CM professions being self-regulated or currently beyond regulation, it is not surprising that patients find it difficult to identify avenues for directing complaints against CM practitioners.^7^ Indeed, the Australian experience of registration of Chinese medicine found that simply incorporating this profession into a well-known regulatory mechanism (state-based registration) resulted in a five-fold increase in complaints, as complaints that had otherwise been lost were able to be appropriately reported.^7^ Such confusion may be confounded by the hesitance of the government to incorporate CM professions and practices into regulatory schemes, even when multiple public government inquiries have made this recommendation. Over a period of more than twenty years, multiple inquiries by three State governments and the Federal government have recommended the government regulation of naturopaths, yet to date there has been no regulatory action.^9^ Traditional traits-based aspects of regulated professions (such as university-level education or access to insurance) have also been extended to many unregistered CM professions, potentially creating greater confusion.^9^

 However, patients finding it difficult to identify avenues for directing complaints against practitioners is not an issue specific to CM practitioners, with studies of other healthcare practitioner groups, such as nurses and allied health professionals, revealing similar findings as this study. In examining the general public’s experiences of reporting healthcare complaints in Sweden, Wessel et al found that the most frequent reason for not making a formal complaint against a healthcare professional was a lack of knowledge about how to complain or even if making a complaint was an option.^15^ In addition, this Swedish study also identified that participants’ lack of knowledge regarding where or to whom they should complain was a significant obstacle that discouraged their willingness to complain. Similarly, Doron et al conducted a study on the complaint patterns of older persons in the healthcare system in Israel found knowledge about their rights to complain and methods of how to make a formal complaint are lacking in this older population with the majority of study participants who had a reason to make a complaint about healthcare services failing to do so.^16^ In reference to the under-reporting of practitioner-induced harm resulting from patients’ lack of knowledge of how or where to complain, evidence from multiple sources suggests that this situation can be markedly improved with the implementation of a clear complaint mechanism.^9^

###  Knowledge About How CM Is Regulated in Australia

 The majority of participants in our study indicated a lack of confidence in knowing where to complain if something went wrong with a herbal or nutritional supplement they had used as well as a lack of knowledge about how herbal and nutritional supplements are regulated. This situation is understandable given the fact that certain CM products are the overlapping or shared jurisdiction of multiple regulators. For example, advertising claims made by CM manufacturers may fall under different jurisdictions, depending on whether the claim has been made in-store, on printed advertising material, on television or online, and whether the product was assessed as a medicinal food or therapeutic good.^9,17^ The confusion about CM product regulation also exists outside Australia. For example, Blendon et al, utilising national opinion surveys to examine US adults’ views on the use and regulation of dietary supplements, identified a substantial percentage of respondents as confused about the role that the government currently plays in regulating dietary supplements.^18^ One important implication of confusing and/or non-existing reporting regimes for CM products is that it limits the availability of adverse events data in relation to CM products and as a result likely leads to significant under-reporting of CM adverse events.^10,19^

###  Future Research Directions

 Amid this complex regulatory and legal landscape, very little is known about the regulatory risks associated with what CM practitioners and manufacturers actually do in their practice, and the impact on consumers; thus impeding the development of effective and workable regulatory and legislative provisions. When regulations of CM are informed by practice (for example, national registration which relies on a system of peer-monitoring), regulation is highly responsive and effective, and in many cases more effective at its goals of protecting the public than many conventional medicine regulations.^20,21^ Regulatory and legislative mechanisms are less effective, and create more regulatory gaps, when they are not specifically focused or informed by CM practice.^9^ It is therefore imperative that researchers take a more active role in prioritising measurement and examination of CM practice – as well as its policy and public health implications – to inform evidence-based regulatory and legislative developments related to the growing role of CM in modern healthcare.

###  Study Limitations

 This study does have some limitations that need to be taken into consideration when interpreting the findings. The information collected through the study questionnaire is based on self-reported information, with no opportunity to validate responses. In addition, the sample was recruited via purposive sampling and is not representative of the wider Australian population, being of a younger age and higher level of education; thus generalisation of study findings needs to be made with caution. Despite these potential limitations, this study is the first to explore the general public’s understanding of CM regulation in Australia, and given the dearth of literature on this topic, may direct important future research and policy discussions on this topic.

## Conclusion

 Despite high levels of public utilisation and support of CM in Australia, the findings of this study clearly highlight a concerning lack of knowledge amongst CM patients and consumers more generally regarding the regulation of CM in Australia. From a policy perspective, it is necessary to seek proactive approaches that target complaint-related knowledge of CM patients through education and advocacy efforts.

## Acknowledgements

 This work was supported by Faculty of Health, University of Technology Sydney, through a Health Futures Development Grant. The research reported in this paper is the sole responsibility of the authors and reflects the independent ideas of the authors alone.

## Ethical issues

 This study has been approved by the Human Research Ethics Committee at the University of Technology Sydney (ETH19-3810).

## Competing interests

 Authors declare that they have no competing interests.

## Authors’ contributions

 DS designed and conducted the study. WP conducted the analyses. DS and JA co-wrote the first draft. JW, CS, and PK provided critical feedback to the draft. All authors discussed the results and contributed to the final manuscript. All authors have approved the final version of the manuscript and the submission to this journal.

## Availability of data and material

 The data used in this study are collected via SurveyGizmo and extracted as an Excel workbook. The data is not available for download.

## References

[1] Adams J, Andrews G, Barnes J, Broom A, Magin P. Traditional, Complementary and Integrative Medicine: An International Reader. London: Palgrave Macmillan; 2012.

[2] Steel A, McIntyre E, Harnett J (2018). Complementary medicine use in the Australian population: results of a nationally-representative cross-sectional survey. Sci Rep.

[3] Australian Institute of Health and Welfare (AIHW). Health Expenditure Australia 2012-13: Analysis by Sector. Health and welfare expenditure series no. 53. Canberra: AIHW; 2014.

[4] Adams J, Sommers E, Robinson N (2013). Public health and health services research in integrative medicine: an emerging, essential focus. Eur J Integr Med.

[5] Wardle J, Adams J, Magalhães RJ, Sibbritt D (2011). Distribution of complementary and alternative medicine (CAM) providers in rural New South Wales, Australia: a step towards explaining high CAM use in rural health?. Aust J Rural Health.

[6] Xue CC, Zhang AL, Lin V, Da Costa C, Story DF (2007). Complementary and alternative medicine use in Australia: a national population-based survey. J Altern Complement Med.

[7] Wardle J, Adams J (2014). Indirect and non-health risks associated with complementary and alternative medicine use: an integrative review. Eur J Integr Med.

[8] Byard RW, Musgrave I, Maker G, Bunce M (2017). What risks do herbal products pose to the Australian community?. Med J Aust.

[9] Wardle J (2017). Complementary health law.

[10] Wardle J, Weir M, Marshall B, Archer E (2014). Regulatory and legislative protections for consumers in complementary medicine: lessons from Australian policy and legal developments. Eur J Integr Med.

[11] Sibbritt D, Millbank J, Stuhmcke A, Kaye M, Karpin I, Wardle J (2016). The failure of contemporary law and regulation to keep pace with growing complementary medicine (CM) use: the significance of examining ‘hidden’gaps in Australia’s current regulatory and legislative infrastructure. Adv Integr Med.

[12] World Health Organization (‎WHO)‎. WHO Global Report on Traditional and Complementary Medicine 2019. WHO; 2019. https://apps.who.int/iris/handle/10665/312342.

[13] Reid R, Steel A, Wardle J, Trubody A, Adams J (2016). Complementary medicine use by the Australian population: a critical mixed studies systematic review of utilisation, perceptions and factors associated with use. BMC Complement Altern Med.

[14] What’s on my medicine label? Therapeutic Goods Administration Website. https://www.tga.gov.au/whats-my-medicine-label. Accessed November 1, 2019.

[15] Wessel M, Lynøe N, Juth N, Helgesson G (2012). The tip of an iceberg? a cross-sectional study of the general public’s experiences of reporting healthcare complaints in Stockholm, Sweden. BMJ Open.

[16] Doron I, Gal I, Shavit M, Weisberg-Yosub P (2011). Unheard voices: complaint patterns of older persons in the health care system. Eur J Ageing.

[17] Harvey KJ, Korczak VS, Marron LJ, Newgreen DB (2008). Commercialism, choice and consumer protection: regulation of complementary medicines in Australia. Med J Aust.

[18] Blendon RJ, DesRoches CM, Benson JM, Brodie M, Altman DE (2001). Americans’ views on the use and regulation of dietary supplements. Arch Intern Med.

[19] Myers SP, Cheras PA (2004). The other side of the coin: safety of complementary and alternative medicine. Med J Aust.

[20] Millbank J, Kaye M, Stuhmcke A, Sibbritt D, Karpin I, Wardle J (2017). Complementary health practitioners disciplined for misconduct in Australia 2010-2016. J Law Med.

[21] Sibbritt D, Kaye M, Millbank J, Stuhmcke A, Wardle J, Karpin I (2018). How are complementary health professions regulated in Australia? an examination of complementary health professions in the national registration and accreditation scheme. Complement Ther Med.

